# Relationship between Blood Stasis Syndrome Score and Cardioankle Vascular Index in Stroke Patients

**DOI:** 10.1155/2012/696983

**Published:** 2012-05-31

**Authors:** Ki-Ho Cho, Kyoo-Pil Kim, Byung-Cheol Woo, Young-Jee Kim, Joo-Young Park, Seung-Yeon Cho, Seong-Uk Park, Woo-Sang Jung, Jung-Mi Park, Sang-Kwan Moon

**Affiliations:** Department of Cardiovascular and Neurologic Disease, College of Oriental Medicine, Kyung Hee University, Seoul 130-702, Republic of Korea

## Abstract

Blood stasis syndrome (BSS) in traditional Asian medicine has been considered to correlate with the extent of atherosclerosis, which can be estimated using the cardioankle vascular index (CAVI). Here, the diagnostic utility of CAVI in predicting BSS was examined. The BSS scores and CAVI were measured in 140 stroke patients and evaluated with respect to stroke risk factors. Receiver operating characteristic (ROC) curve analysis was used to determine the diagnostic accuracy of CAVI for the diagnosis of BSS. The BSS scores correlated significantly with CAVI, age, and systolic blood pressure (SBP). Multiple logistic regression analysis showed that CAVI was a significant associate factor for BSS (OR 1.55, *P* = 0.032) after adjusting for the age and SBP. The ROC curve showed that CAVI and age provided moderate diagnostic accuracy for BSS (area under the ROC curve (AUC) for CAVI, 0.703, *P* < 0.001; AUC for age, 0.692, *P* = 0.001). The AUC of the “CAVI+Age,” which was calculated by combining CAVI with age, showed better accuracy (0.759, *P* < 0.0001) than those of CAVI or age. The present study suggests that the CAVI combined with age can clinically serve as an objective tool to diagnose BSS in stroke patients.

## 1. Introduction

Blood stasis syndrome (BSS) is defined by retardation or cessation of the blood flow and is regarded as the cause or product of many chronic diseases in traditional Asian medicine. Traditionally, the diagnosis of BSS depended on subjective diagnostic methods such as inspection and palpation of the patient [1]. In 1983, Terasawa et al. developed a diagnostic criterion for “Oketsu” (BSS in Japanese), which comprises numerous symptom scores and is among the most widely accepted BSS scores [2–4]. Recent reports have suggested that BSS is correlated with haemorheologic changes such as the deterioration of erythrocyte deformability, elevation of blood viscosity, and acceleration of erythrocyte aggregation, as well as microcirculatory dysfunction [3, 4]. However, the diagnosis of BSS must still be complemented by scientific and objective methods.

BSS is considered to be closely related to senile diseases such as atherosclerosis, ischaemic heart disease, and stroke [5], as well as rheumatoid arthritis, Behçet's disease, hyperuricaemia, and various inflammatory conditions [3]. With regard to atherosclerosis, the carotid intima-media thickness (IMT) has been reported to be closely correlated with the BSS [6], and, besides, treatment of BSS has received recent attention as a therapeutic principle in traditional Chinese medicine (TCM) for atherosclerosis [5]. In addition, atherosclerosis is known to be correlated with arterial stiffness [7]. The cardio-ankle vascular index (CAVI) is thought to be a noninvasive and useful method to evaluate the arterial stiffness [8], and it has been used to estimate the extent of atherosclerosis [7, 9]. Therefore, the CAVI is likely to provide supplementary information for the diagnosis of BSS. However, to our knowledge, no study has addressed the correlation between the CAVI and the BSS score.

The purpose of this study was to assess the relationship between the BSS score and the CAVI in stroke patients and to estimate the role of the CAVI as a diagnostic tool for BSS using the ROC curve.

## 2. Methods

### 2.1. Subjects

From April 2006 to May 2007, 810 patients who were hospitalized with ischaemic or haemorrhagic stroke diagnosed by brain CT or MRI were recruited in the Kyung Hee University Oriental Medicine Hospital. We excluded patients in the acute stage within 10 days after stroke onset and whose BSS scores could not be assessed because of impaired cognitive function. The remaining 140 patients were included in this study. Written informed consent was obtained from all patients after the Institutional Review Board of Kyung Hee Oriental Medicine Hospital approved the study protocol.

### 2.2. Estimation of Blood Stasis Syndrome Score

For the evaluation of the BSS score, we used the “Oketsu” scoring system, with the diagnostic criteria developed by Terasawa et al. (Table 1) [2]. This BSS scoring system consists of 17 inquiries with 3 scales of points that are determined after extensive multivariate analyses; the resultant score in this system has been reported to have a quantitative relationship with haemorheology data [3]. According to the BSS score, patients were classified into 2 categories: a “non-BSS” state (BSS score ≤ 20) and a “BSS” state (BSS score > 20).

### 2.3. Measurement of the CAVI

The arterial stiffness was assessed by determining the CAVI (VaSera VS-1000; Fukuda Denshi, Tokyo, Japan). In accordance with the device manufacturer's instructions, the subjects rested in the supine position for at least 10 min before measurements were obtained. The cuffs were attached to the 4 extremities, and electrocardiographic electrodes were attached to the upper extremities. A microphone was placed on the sternal angle for phonocardiography. The CAVI was automatically calculated using a waveform analyser in the VaSera VS-1000 [10, 11]. The complete measurement of all CAVIs was usually completed in <5 min.

### 2.4. Clinical Assessments

Information regarding the potential vascular risk factors for each subject, including tobacco smoking, history of MI, and the use of antihypertensive and hypoglycaemic agents, was recorded from patient interviews and medical records. For current smoking, subjects must have reported smoking at least 100 cigarettes over their lifespan and a current smoking frequency of occasional or every day, at the time of interview. History of myocardial infarction (MI) and left ventricular hypertrophy (LVH) were confirmed by reviewing the medical records or by electrocardiography. Hypertension was defined as the presence of a history of hypertension, a systolic blood pressure (SBP) of ≥140 mmHg, or a diastolic pressure of ≥90 mmHg. SBP as a variable for analysis was recorded from the higher brachial SBP, which was checked during the measurement of the CAVI. DM was diagnosed if the subject was currently undergoing treatment with insulin or oral hypoglycaemic agents, or if the fasting blood glucose level was ≥140 mg/dL. Blood was drawn for biochemical analyses, including estimation of serum level of total cholesterol and creatinine following an overnight fast in the initial study.

### 2.5. Statistical Analysis

The data were expressed as case numbers or means ± standard deviation (SD). To compare the means of continuous variables, Student's *t*-test was applied. Categorical variables were analysed using a chi-square analysis or Fisher's exact test. Correlations between continuous variables were determined using the Pearson's correlation coefficient. The variables found to be associated with BSS were further tested by a multiple logistic regression analysis to investigate the independent factors for BSS. To assess the ability of each variable to discriminate the BSS, the areas under the receiver operating characteristic (ROC) curves (AUC) were calculated. In addition, the asymptotic 95% confidence interval (CI) and *P* values under the null hypothesis (true area = 0.50) were calculated. An AUC of >0.9 was considered excellent; 0.8–0.9, very good; 0.7–0.8, good; 0.6–0.7, average; <0.6, poor [10, 12]. Statistical significance was defined as *P* < 0.05. All statistical analyses were performed with SPSS version 12.0 (SPSS Inc., Chicago, IL, USA), whereas the ROC curves were calculated by MedCalc version 12.1.4.0.

## 3. Results

Characteristics of the participants are shown in Table 2. Of 140 stroke patients, 118 (84.3%) were diagnosed with BSS. The BSS group was found to have higher CAVI (*P* < 0.01), age (*P* < 0.01), and SBP (*P* = 0.04) than the non-BSS group. However, other variables, including stroke risk factors, did not statistically differ between individuals with and without BSS.

**Figure 1:**
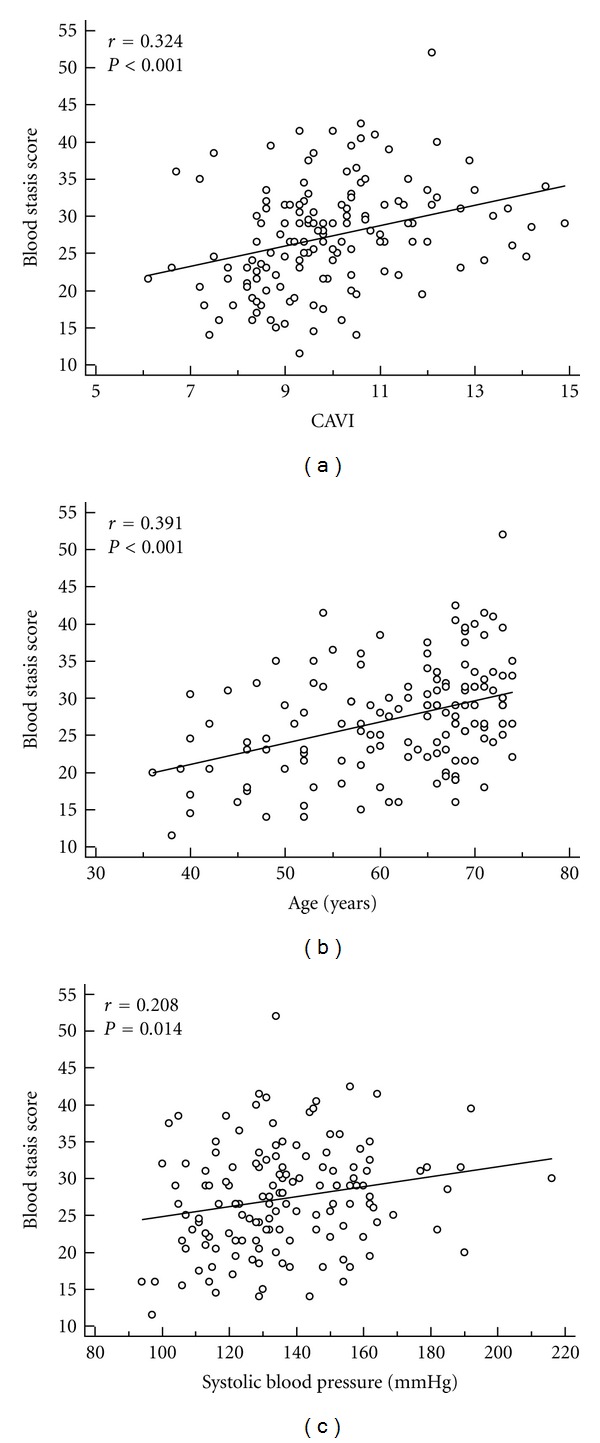
The correlation between the blood stasis score and the cardio-ankle vascular index (CAVI) was significant (*n* = 140, *r* [Pearson's correlation coefficient] = 0.324, *P* < 0.001) in stroke patients (a). In addition, the age (b) and systolic blood pressure (c) were significantly correlated with the blood stasis score (*r* = 0.391, *P* < 0.001; *r* = 0.208, *P* = 0.014, resp.).

The correlation between the BSS scores and CAVI was significant (*r* = 0.324, *P* < 0.001) in stroke patients. In addition, the age and SBP were significantly correlated with the BSS scores (*r* = 0.391, *P* < 0.001; *r* = 0.208, *P* = 0.014, resp.) (Figure 1).

A multiple logistic regression analysis showed that the CAVI was a significant associate factor for BSS (OR 1.55, *P* = 0.032) after adjusting for the age and SBP, both of which were not significant in the model (Table 3).

**Figure 2:**
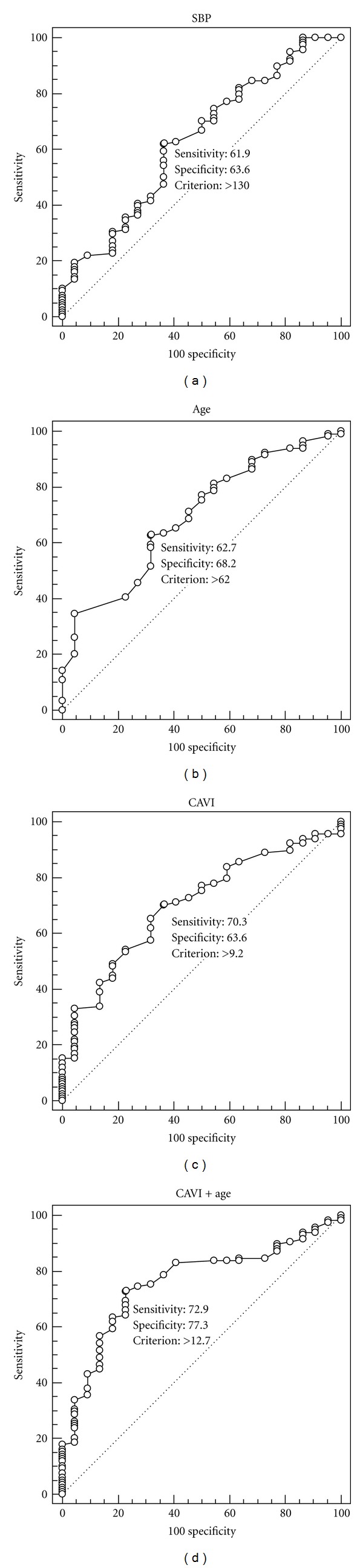
The diagnostic accuracy of the systolic blood pressure (SBP), age, CAVI, and CAVI+Age for predicting blood stasis syndrome (BSS) in stroke patients. The ROC curves depicted that the CAVI and age showed modest diagnostic utility for BSS with the CAVI+Age indicating good diagnostic accuracy, while SBP provided poor diagnostic utility. In each graph, the solid diagonal line was the line of no discrimination (area = 0.5), and the optimal cut-off points were indicated on the curves.

ROC curves were generated for CAVI, age, and SBP to determine their possible diagnostic utility for distinguishing the BSS groups from the non-BSS groups (Figure 2). The CAVI and age showed modest utility with ROC curves that were higher and shifted more to the left than those of SBP, which showed poor utility. Based on the area under the ROC curve (AUC), by which the accuracy of the test is measured, the AUC of the CAVI and age showed average accuracy (0.703 and 0.692, resp.), with no significant difference between these values. However, the SBP indicated an AUC of 0.630, which did not reject the null hypothesis (true area = 0.50) (Table 4). To find a better discriminator of BSS, a new variable “CAVI+Age” was calculated by combining the CAVI with age as follows: the age was categorized into ages <40, 40–49, 50–59, 60–69, and ≥70; then converted into 1, 2, 3, 4, and 5, respectively; finally added to CAVI scores. The AUC of the “CAVI+Age” (0.759) showed better accuracy than those of the CAVI or age although there was no significant difference among those values (Table 4) (Figure 2).

To determine the optimal threshold for the diagnosis of BSS, the intersection point between the sensitivity and the 100-specificity curves of the CAVI, age, and “CAVI+Age” was used. The optimal cut-off points for the CAVI, age, and “CAVI+Age” were 9.2, 62 years, and 12.7, respectively. Using the threshold of 9.2 for the CAVI, 62 years for the age, and 12.7 for the “CAVI+Age”, the sensitivities were 70.3%, 62.7%, and 72.9%, respectively, and the specificities were 63.6%, 68.2%, and 77.3%, respectively (Figure 2).

## 4. Discussion

Ancient Chinese medical texts describe a disorder of the blood circulation, which causes various symptoms such as BSS, reduced blood flow, and cessation of flow. This phenomenon is commonly observed in Asian countries but termed differently as “Yu Xue” in Chinese, “Eohyul” in Korean, or “Oketsu” in Japanese. For the diagnosis of BSS, Terasawa et al. developed a diagnostic criterion of “Oketsu,” which has become one of the most widely accepted methods for BSS scoring [2, 3]. However, the diagnosis of BSS still needs to be complemented by scientific and objective methods [3]. In the present study, the ROC analysis indicated that the AUC of the CAVI and age showed fair diagnostic accuracy for BSS. Furthermore, The AUC of the “CAVI+Age” showed better accuracy than those of the CAVI or age.

BSS has been reported to be closely correlated with atherosclerosis [6], as well as disorders of the peripheral microcirculation, rheumatoid arthritis, systemic lupus erythaematosus (SLE), disseminated intravascular coagulation (DIC), and various allergic responses [3]. With regard to the relationship between BSS and atherosclerosis, Lei et al. reported that the carotid IMT was closely correlated with BSS in patients with dyslipidaemia. In addition, Ma and Chen indicated in a review paper that the treatment of BSS has received recent attention as a therapeutic principle in TCM for atherosclerosis [5]. In this regard, a traditional Chinese drug to relieve BSS, the Xuefuzhuyu pill, was reported to be beneficial to retard the progress of atherosclerosis [13, 14]. In Japan, Keishi-bukuryo-gan-ryo, which is one of the most important prescriptions for improving BSS, has been reported to prevent the progression of atheromatous plaque by strengthening the antioxidant defence system [15] and exerting a protective effect on the endothelium [11]. These studies support the relationship between BSS and atherosclerosis. Therefore, the diagnostic methods for atherosclerosis are likely to complement the diagnosis of BSS in an objective manner.

Atherosclerosis is known to be correlated with arterial stiffness [16] and the progression of coronary artery sclerosis [7, 16]. The aortic (carotid-femoral) pulse wave velocity (cfPWV) is a well-established index of central arterial stiffness. However, one drawback of this index is that the accuracy of cfPWV measurements by Doppler imaging or tonometry depends greatly on the skill and experience of the practitioner. The recent introduction of the volume plethysmographic method allows the measurement of brachial ankle pulse wave velocity (baPWV) and the cardio ankle vascular index (CAVI) with minimal technical skill [17]. Furthermore, the CAVI, which is independent of the blood pressure, can clinically serve as a predictive marker of the extent of coronary artery disease (CAD) and has been reported to increase the diagnostic performance of CAD over baPWV [7, 18]. The CAVI was also reported to be a useful clinical marker for evaluating atherosclerosis and arteriolosclerosis in patients with essential hypertension [19]. Therefore, the CAVI is thought to be an easy, noninvasive and useful method to estimate the extent of atherosclerosis, and it is likely to provide supplementary information on the diagnosis of BSS.

In the present study, we used ROC analysis, which is a useful tool to evaluate the performance of diagnostic tests [20] to evaluate the diagnostic performance of the CAVI for BSS diagnosis. Generally, an ROC curve is a plot of sensitivity on the *y* axis against “1-specificity” on the *x* axis for varying values of the threshold *t*. The AUC provides an overall summary of the diagnostic accuracy. The AUC equals 0.5 when the ROC curve corresponds to random chance, and 1.0 under conditions of perfect accuracy. When the estimated AUC is <0.5, the test is less predictive than chance [20]. In this study, the AUC of the CAVI and age were 0.703 and 0.692, respectively, thereby demonstrating average diagnostic accuracy for predicting BSS in stroke patients. Although there was no significant difference between these outcome measures in the comparison of the AUC, the multiple logistic regression analysis showed that the CAVI was a significant factor for BSS after adjusting for the age and SBP, both of which were not significant in the model. Thus, we suggest that the CAVI might be more valuable for discerning the presence of BSS in stroke patients than the age.

The CAVI has been reported to correlate with age [7]. In humans, aging is a considered a strong contributing factor for atherosclerosis. With aging, the degenerated and decreased elastic fibres of the media in the large arterial wall can lead to an increase in collagen fibres and matrix. Such changes increase the aortic stiffness. It has been also reported that the cfPWV and baPWV increased with age, which was associated with an increase in aortic stiffness [21, 22]. Therefore, it is conceivable for age to have a fair diagnostic accuracy for BSS diagnosis, considering the relationship between CAVI and BSS score. Furthermore, we found that the AUC of the combined variable “CAVI+Age” demonstrated better accuracy than those of the CAVI or age, which suggests that the CAVI combined with age might be a better discriminator of BSS than the CAVI alone.

The present study has several limitations. First, the ROC curve was difficult to interpret because of the small sample size. The ROC analysis in the present study thus represents a preliminary trial that should be extended to a larger cohort. In addition, the definition of cutoff values of the CAVI for discerning the presence of BSS may be required for every age group. Second, our results may be disease-specific, because the subjects comprised only stroke patients. Thus, validation studies with larger cohorts, methodological improvement, and strictly defined protocols are necessary for future studies.

In conclusion, our results suggest that the CAVI combined with age can clinically serve as an objective tool to diagnose BSS in stroke patients. The present study sheds light on traditional medical concepts from the viewpoint of modern science and medicine.

## Figures and Tables

**Table 1:** Diagnostic criteria for blood stasis syndrome (BSS).

Symptom	Score	
	Male	Female
Dark-rimmed eyes	10	10
Areas of dark pigmentation of facial skin	2	2
Rough skin	2	5
Livid lips	2	2
Livid gingival	10	5
Livid tongue	10	10
Telangiectasis/vascular spiders	5	5
Subcutaneous hemorrhage	2	10
Palmar erythema	2	5
Resistance and tenderness on pressure of the left paraumbilical region	5	5
Resistance and tenderness on pressure of the right paraumbilical region	10	10
Resistance and tenderness on pressure of the umbilical region	5	5
Resistance and/or tenderness on pressure of the ileocecal region	5	2
Resistance and/or tenderness on pressureof the sigmoidal region	5	5
Resistance and/or tenderness on pressureof the subcostal region	5	5
Hemorrhoids	10	5
Dysmenorrhea	—	10

A total score larger than 20 is diagnosed as a BSS and that not exceeding 20 is diagnosed as a non-BSS. Mild symptoms are designated by half points.

**Table 2:** Characteristics of the study participants.

Variables	Blood stasis syndrome		*P*-value
	No (*n* = 22)	Yes (*n* = 118)	
Male sex, %	50.0 (11)	47.5 (56)	0.83
Mean age, y	56.0 (10.6)	62.8 (9.3)	0.003
Age, %			0.004
<40	4.5 (1)	1.7 (2)	
40–49	27.3 (6)	9.3 (11)	
50–59	22.7 (5)	20.3 (24)	
60–69	40.9 (9)	42.4 (50)	
≥70	4.5 (1)	26.3 (31)	
Total cholestrol, mg/dL	176.14 (36.73)	179.79 (39.69)	0.69
Systolic blood pressure, mmHg	127.32 (20.11)	137.97 (21.83)	0.04
Serum creatinine, mg/dL	0.69 (0.25)	0.73 (0.30)	0.53
Height, cm	161.60 (7.58)	161.34 (8.49)	0.90
CAVI	9.01 (1.12)	10.09 (1.72)	0.006
History MI, %	0 (0)	0 (0)	—
LVH, %	4.5 (1)	5.9 (7)	0.80
Diabetes, %	18.2 (4)	30.5 (36)	0.24
Hypertension, %	63.6 (14)	65.3 (77)	0.88
Current smoke, %	22.7 (5)	17.8 (21)	0.59
Stroke type			0.89
Infarction, %	81.8 (18)	80.5 (95)	
Hemorrhage, %	18.2 (4)	19.5 (23)	

Data are mean (SD) or % (*n*). *P* value represents significance of differences between groups using *t*-test, *χ* ^2^ test. CAVI: Cardio-ankle vascular index; MI: Myocardial infarction; LVH: left ventricular hypertrophy.

**Table 3:** Multiple logistic regression analysis of associated variables for blood stasis syndrome.

Variables	Odds ratio*	95% Confidence interval	*P* value
CAVI	1.55	1.04–2.32	0.032
Age	1.04	0.99–1.10	0.090
SBP	1.01	0.99–1.04	0.267

CAVI: Cardio-ankle vascular index; SBP: Systolic blood pressure.

*Adjusted for all the other variables shown in this table.

**Table 4:** Area under the receiver-operator characteristic (ROC) curve for the CAVI+Age, CAVI, age, and systolic blood pressure as discriminators of blood stasis syndrome among stroke patients.

Variables	Area under ROC curve	95% Confidence interval	*P* value
CAVI + Age	0.759	0.680–0.827	<0.0001
CAVI	0.703	0.620–0.777	0.0003
Age	0.692	0.609–0.767	0.0010
SBP	0.630	0.545–0.710	0.0522

CAVI: Cardio-ankle vascular index; SBP: Systolic blood pressure.
